# Clinical Characteristics and Current Practice of Endovascular Revascularization in Aorto-Iliac, Femoropopliteal and Infra-Popliteal Lower Extremity Artery Disease—Insights from the RECCORD Registry

**DOI:** 10.3390/jcm11206074

**Published:** 2022-10-14

**Authors:** Jacqueline Stella, Jürgen Stausberg, Michael Lichtenberg, Ulrich Hoffmann, Nasser M. Malyar

**Affiliations:** 1 Department of Cardiology I—Coronary and Peripheral Vascular Disease, Heart Failure, University Hospital Muenster, Cardiol, 48149 Muenster, Germany; 2 Independent Researcher, 45276 Essen, Germany; 3 Vascular Center, Arnsberg Clinic, 59759 Arnsberg, Germany; 4 Division of Vascular Medicine, Medical Clinic and Policlinic IV, Hospital of the Ludwig-Maximilians-University, 80336 Munich, Germany

**Keywords:** endovascular revascularization, lower extremity artery disease, aorto-iliac, femoropopliteal, infrapopliteal

## Abstract

Background: Endovascular revascularization (EVR) is a pillar of therapeutic management in patients with symptomatic lower extremity artery disease (LEAD). Due to lack of scientific evidence, the approach of EVR type and the devices used at the different anatomic vascular segments of the lower limbs vary substantially between operators and centers. We analyzed data from the RECcording COurses of vasculaR Diseases (RECCORD) registry to assess the current real-world EVR treatment patterns in relation to anatomic vascular segments in symptomatic LEAD patients in Germany. Patients and Methods: RECCORD is an ongoing, prospective, multicenter, all-comers and entirely web-based registry platform. Baseline demographic and periprocedural data of patients undergoing EVR for symptomatic LEAD were assessed and performed EVRs were grouped according to the intervened anatomic vascular segment. We analyzed four EVR groups comprising either the aorto-iliac, femoropopliteal, or infrapopliteal segments (all these EVRs with or without a further intervention in another anatomic segment) or the infrapopliteal segment alone. Results: A total of 2210 EVR segments (in 1639 patients) were analyzed. Of those 616 (27.9%) were aorto-iliacal, 1346 (60.9%) femoropopliteal, 248 (11.2%) infrapopliteal and 104 (4.7%) only infrapopliteal segments. Aorto-iliac EVR was associated with younger age, smoking, claudication and simple lesions, while the distal infrapopliteal EVRs were related to advanced age, diabetes, multiple comorbidities, limb threatening ischemia and complex lesions. The use of different EVR devices at the aorto-iliac, femoropopliteal, infrapopliteal and only infrapopliteal segments were: only ballon-angioplasty: 8.3%, 12.9%, 58.1% and 63.5%; stenting: 82.3%, 45.3%, 16.9% and 12.5%; drug-coated balloon: 11.2%, 55.0%, 19.4% and 19.2%. Conclusion: The RECCORD registry data demonstrate that in LEAD clinical and lesion characteristics are related to anatomic vascular segments. Despite the clear relationship between vascular segments and the current use of device types, prospective, segment-specific clinical studies are warranted to establish a consistent, evidence-based path for EVR in LEAD.

## 1. Introduction

Endovascular revascularization (EVR) is a pillar of therapeutic management in patients with symptomatic lower extremity artery disease (LEAD). Increasing procedural experience alongside development of dedicated technical devices in the last 2 decades resulted in excellent technical success rates beyond 90%, even in the most challenging and complex lesions. The long-term success of EVR, however, is hampered by restenosis and re-occlusion due to the chronic progressive nature of atherosclerosis. Due to differences in anatomic and physiologic properties of the arteries, the rates of restenosis/re-occlusion vary at different anatomic levels of the lower limbs, i.e., in aorto-iliac, femoropopliteal and in infrapopliteal arteries. Influenced by patient’s comorbidities, by the severity of arteriosclerotic disease, lesion length, calcification and lesion morphology as well as by the technical devices used for EVR, the long-term patency deteriorates markedly from the aorto-iliac to the distal segments of the infrapopliteal, crural arteries [1]. Continuous development and improvement of dedicated tools and devices entering the EVR arena for treatment of LEAD occur at a speed which outperforms the evolvement of the underlying scientific evidence. In fact, an evidence-based approach of the technical devices used for EVR in relation to anatomical levels is lacking. The decision which device to use for which lesion and at which anatomic level depends on the preference of the operator.

We aimed to depict the current practice of EVR techniques and used devices in relation to different anatomic regions of the lower extremities in the RECcording COurses of vasculaR Diseases (RECCORD) registry.

## 2. Materials and Methods

This study utilized data from the RECCORD registry, which comprises all patients undergoing EVR for symptomatic peripheral arterial disease (PAD) located from the aorto-iliac bifurcation to the distal pedal artery segments in Germany between 1 February 2019 and 30 March 2020. RECCORD was set up by the German Society of Angiology—Society for Vascular Medicine (DGA) and is a prospective, multicenter, all-comers registry. Patients’ demographic characteristics including history of cardiovascular and limb events, risk factors, comorbidities, medication, peri- and postprocedural data including complications and planned and unplanned clinical visits were recorded in an entirely web-based registry platform. The rationale, design and detailed methods of the RECCORD registry has previously been published [2,3]. The registry was approved by the ethic committee of the Medical Faculty of the Ludwig-Maximilian University of Munich and informed consent is obtained from every patient prior to inclusion in the study. Exclusion criteria were patients undergoing hybrid (surgical and endovascular) and/or surgical intervention for the treatment of arterial occlusive disease as well as endovascular interventions not aimed at treating arterial occlusive disease.

Per definition, the vascular bed of the lower extremities was divided in three anatomic segments along the lines of the 2015 recommendation of the German guideline on the diagnosis and treatment of peripheral artery disease: the aorto-iliac (from the aorto-iliac bifurcation to the common femoral artery), the femoropopliteal (from the femoral bifurcation to the distal part of the popliteal artery) and infrapopliteal (from the anterior tibial artery/tibio-peroneal trunc bifurcation to the pedal plantar arch) segments. According to these anatomic levels, we divided the entire study population into four different groups: the aorto-iliac, femoropopliteal, and infrapopliteal group (which included all EVRs with an involvement of the respective anatomic segment and a possible involvement of one or two other segments if the revascularization procedure required an intervention in more than one segment), and only infrapopliteal group (which included EVRs performed exclusively in the infrapopliteal segment). The latter group was defined because patients with exclusively lesions of the crural arteries constitute a distinct LEAD subpopulation having a high comorbidity score (often diabetes, renal and heart failure) with characteristic complex lesion morphology resulting in unfavorable short and long-term outcome.

We included 1639 patients in the analysis with a total of 2210 EVRs. We mainly performed the analysis according to the anatomical segments, therefore we analyzed 1898 EVR in different anatomical segments. The difference in the total amount of EVRs (*n* = 2210) and the amount of EVRs in the different anatomical segments (*n* = 1898) was caused by patients with an intervention performed in both legs but the same anatomic segment (e.g., right and left aorto-iliac revascularization). Those interventions accounted for two EVRs but only one EVR according to the anatomic segment.

Clinical characteristics were described as numbers and percentages for categorical variables, and as means with standard deviations for continuous variables. Continuous variables were compared using the two-sided unpaired *t*-test and Kruskal-Wallis H test, categorical values using chi-squared or Fisher’s exact tests. All data were analyzed using IBM SPSS Statistics 25 (IBM, Armonk, NY, USA). For all tests, a *p*-value < 0.05 was considered significant.

## 3. Results

### 3.1. Baseline Characteristics

During a period of 14 months, 1639 patients (summing up to 2210 revascularized segments) were enrolled at 24 vascular centers in Germany. Baseline demographic and clinical characteristics of the study population for the four different groups representing the different anatomic segments are summarized in Table 1.

Of the entire study population 565 (34.5%) were female and 1074 (65.5%) were male. The proportion of male gender continuously increased from the proximal to the distal segments of the lower limb arteries (aorto-iliac 64.1% vs. femoropoliteal 65.2% vs. infrapopliteal 68.4% vs. only infrapopliteal 73.7%). Mean age rose from 66.9 years in the aorto-iliac segment group to 76.0 years in the only infrapopliteal group. Likewise, the proportion of octogenarians increased from the aorto-iliac towards infrapopliteal vascular segments (aorto-iliac 8.2% vs. femoropopliteal 17.5% vs. infrapopliteal 38.5% vs. only infrapopliteal 43.2%). 

The overall study population exhibited a high burden of classic cardiovascular risk factors and comorbidities as illustrated in Table 1. While the prevalence of diabetes increased from the aorto-iliac (28.3%) to only infrapopliteal segments (60.0%), the prevalence of active smoking conversely decreased from 64.3% at the aorto-iliac to 16.8% at only infrapopliteal segments. Coronary artery disease, chronic heart failure, renal failure and polyneuropathy showed a rising trend from the aorto-iliac downstream to the infrapopliteal segments.

Among the entire study population, 4.0% of all patients had a history of amputation prior to the index intervention with the lowest fraction in the aorto-iliac (2.0%) and the highest fraction in the only infrapopliteal subgroup (17.9%). Previous EVR was documented for 35.9% of aorto-iliac, 40.8% for femoropopliteal, 41.1% for infrapopliteal and 30.8% for only infrapopliteal interventions, respectively. Previous surgical interventions were reported for 8.3% of aorto-iliac, 8.7% of femoropopliteal, 8.9% of infrapopliteal and 7.7% of only infrapopliteal segments (Table 1).

The clinical stages of LEAD as indicated by the Rutherford categories for the four different anatomic groups are also presented in Table 1. The vast majority of EVR of the aorto-iliac segment was performed due to claudication (>90%), whereas EVR due to chronic limb-threatening ischemia (CLTI) constituted a minor portion (8.6%) in this anatomic segment. In only infrapopliteal segments, however, this ratio was inversed with 74.0% of patients being at the stage of CLTI and 26.0% at claudication.

### 3.2. Procedural Characteristics

Of all EVR segments (*n* = 2210) of the lower extremities, 616 (27.9%) were performed at aorto-iliac, 1346 (60.9%) at femoropopliteal, 248 (11.2%) at infrapopliteal without or with proximal (+aorto-iliac and/or femoropopliteal) segments and 104 (4.7%) exclusively at only infrapopliteal segments. Multisegmental EVR at a single procedure was common in aorto-iliac (44.9%) and infrapopliteal (59.3%) level, yet less frequent in femoropopliteal (21.5%) and rarely performed at only infrapopliteal segments (1.1%, Table 2).

The procedural characteristics of EVR are presented in Table 2. Regarding target lesions, the proportion of total occlusions increased from the aorto-iliac (16.4%) to the only infrapopliteal segments (57.7%). Also, aorto-iliac lesions were shorter than infrainguinal ones (lesion length < 10 cm in 87.0% of aorto-iliac vs. 40.4% in only infrapopliteal segments).

With regard to endovascular treatment details, the use of plain-old balloon angioplasty (POBA) as the only treatment modality increased from 8.3% at the aorto-iliac level to 63.5% at the only infrapopliteal level. Conversely, adjunctive stenting decreased from 82.3% at the aorto-iliac segments to 12.5% at the only infrapopliteal segments. Drug-coated balloons (DCB) were predominantly used at the femoropopliteal segments (55.0%) while its use was less frequent at the aorto-iliac (11.2%) and only infrapopliteal segments (19.2%). Debulking devices (atherectomy, rotational thrombectomy) were used in 1.9%, 12.4%, 9.7% and 9.6% of cases at the aorto-iliac, femoropopliteal, infrapopliteal and only infrapopliteal segments, respectively. The number of stents and DCBs used for a single EVR procedure is presented in Table 2. 

### 3.3. Peri-Procedural and in-Hospital Complications

Peri-procedural complications are presented in Table 3. A total of 94 (5.0%) interventions suffered from complications with 48 (2.5%) regarding the access site and 51 (2.7%) being procedure related. The most common complications were post-interventional pseudoaneurysm (*n* = 36; 38.3% of all complications) and distal embolization (*n* = 36, 38.3% of all complications). Access site as well as procedure related complications occurred at higher rates at EVR at the distal segments involving the infrapopliteal segments. Complications requiring surgical or endovascular intervention were low for all anatomic regions ranging from 0.4% for femoropopliteal EVR to 1.1% for only infrapopliteal EVR. Severe periprocedural clinical events were rare: Two patients died during the in-hospital stay due to unknown causes, three patients had a myocardial infarction (1 femoropopliteal EVR, 2 infrapopliteal EVR), one patient undergoing femoropopliteal EVR suffered from a stroke.

### 3.4. Baseline and Postinterventional Medication

The medication at baseline and after the first, i.e., after the index-EVR, for all vascular segments is presented in Table 4. About three quarters of all EVR-patients were on some types of lipid lowering agents at baseline. The rate increased significantly after EVR in all subgroups (*p* < 0.01). More than 80% received platelet inhibitors before EVR and likewise this rate also increased to >95% in all groups. Use of ACE inhibitors and beta-blockers did not change after EVR. Compared to baseline values, the prescription rate of direct oral anticoagulants increased in all subgroups with a continuous increment from proximal to distal EVR segments.

## 4. Discussion

A continuous ageing population alongside with an increase in atherosclerotic vascular risk factors, such as diabetes, led to an increase in prevalence of symptomatic LEAD in the last decades. The evolution and progress of endovascular techniques and endovascular device technology for the management of symptomatic LEAD over the last decades resulted in an increase in EVR, with a subsequent reduction in open surgical revascularization procedures. However, until now, no evidence-based path or specific algorithm for the diverse types of EVR in relation to different vascular segments exists. In fact, due to the lack of evidence and guidelines-recommendations, a potpourri of EVR devices are utilized in all vascular segments and the choices of devices are mainly based on the preference of the operators. The data from the all-comers RECCORD registry illustrate well the current practice of EVR at the different anatomic levels in patients with LEAD in Germany.

### 4.1. Demographic Characteristics and Comorbidities in Relation to Anatomic Segments

The advanced age and a high proportion of comorbidities depict the typical high-risk LEAD population in the RECCORD registry. However, there were differences in baseline characteristics and in the frequencies of comorbidities with relation to specific anatomic segments: Except for a high prevalence of active smoking (64.3%), patients with aorto-iliac EVR were younger, had the lowest prevalence of comorbidities such as diabetes, heart and renal failure, were treated for claudication (>90%) rather than for CLTI and showed less complex lesions (i.e., shorter lesion length and less total occlusions). There was a clear trend—from the aorto-iliac towards only infrapopliteal segments—in increasing prevalence of diabetes (28.3% vs. 60.0%), heart failure (5.4% vs. 15.8%), renal failure (16.6% vs. 49.5%), polyneuropathy (4.6% vs. 17.9%) and CLTI stage of LEAD (8.6% vs. 74.0%). Diabetes is a common aggravating co-morbidity in patients with LEAD and predisposes to complex crural disease [4,5], which corresponds well to the findings in the RECCORD registry: Diabetes mellitus was associated with involvement of infrapopliteal arteries and less related to aorto-iliac and femoropopliteal involvement.

The lower frequencies of comorbidities as well as the less complex lesion characteristics of the aorto-iliac segments may-at least partially-explain the observed favorable long-term outcome of aorto-iliac EVR when compared to the femoropoliteal and crural segments.

### 4.2. Procedural Characteristics in Relation to Anatomic Segments

As illustrated in Table 1 and Table 2, >90% of aorto-iliac lesions were treated for claudication and >80% for chronic or acute-on-chronic symptoms. The lesion complexity as indicated by the nature of the lesion (83.6% stenosis) and the lesion length (87% of lesions < 10 cm) clearly increased from the aorto-iliac towards infrapopliteal segments (44.4% and 50.0%, respectively).

Femoropopliteal was the dominant EVR segment (60.9%) while EVR exclusively at infrapopliteal segments were rare (4.7%). The femoropopliteal segment is prone to arteriosclerotic disease, as it covers two major flexion points in the hip and knee. In addition, the superficial femoral artery, which is the longest artery and endures multiple external forces with leg movement, also goes through the adductor canal, a tight space enclosed by multiple muscles of the legs leading to compression and extension during activity [6]. Such anatomic-mechanical forces make the femoropopliteal segment vulnerable and prone for atherosclerotic manifestation. 

Regarding EVR modality, substantial differences were observed between different anatomic segments: Aorto-iliac lesions were mainly treated by deployment of stents (82.3%), while POBA (8.3%) and DCB (11.2%) played a minor role. The dominant segment for DCB use was the femoropopliteal segment with adjunctive DCB use in 55.0% of all procedures. Stents were used in 45.3% of all femoropopliteal EVR indicating still a major role for stenting in the management of femoropopliteal segments despite the advocated “leave-nothing-behind” concept. It is also remarkable that the concern of paclitaxel-induced late mortality which entered the EVR panel by the Katsanos analysis [7] seems to still affect the decision for DCB use at femoropopliteal segments. Given the evidence for benefit of DCB-use at femoropopliteal segments, one would expect a higher rate of DCB-use at these segments than the practiced 55%. Conversely to aorto-iliac segments, only infrapopliteal segments are mainly treated by POBA (63.5%), while DCB (19.2%) and stenting (12.5%) are used less frequently.

### 4.3. Periprocedural Complications

Given the high-risk profile and a high comorbidity score of the RECCORD population, the total periprocedural complication rate of 5.0% was at an acceptable level. Particularly, severe complications requiring a surgical or an endovascular intervention were rare, i.e., ≤1%. Access site- and procedure-related complications were equally distributed, with pseudoaneurysm and peripheral embolism as the main complications of access site- and procedure-related complications, respectively. The higher complication rates involving infrapopliteal segments might be associated with the higher comorbidity score and the more complex lesions in this subcohort. In general, the complication rate reported from the RECCORD registry is within the scope of previously recorded periprocedural complications in LEAD populations. Boc et al. described distal embolization with an overall incidence of 0.9%, but found the highest incidence in the femoropopliteal region with and without involvement of the crural arteries [8]. Ortiz et al. found 3.5% access site related complications [9]. Bhardwaj et al. found a periprocedural incidence rate of major bleeding in 4.1% of patients with aorto-iliac and multi-lesion EVR being associated with higher bleeding events [10].

### 4.4. Secondary Prevention and Medication

According to current guideline recommendations [11,12,13], all patients with symptomatic LEAD should receive a comprehensive secondary prevention program consisting of optimally controlled cardiovascular risk profile, life-style modification, nicotine abstinence, a structured exercise training and evidence-based pharmacotherapy in order to reduce the extensively high rates of adverse cardiac and limb events. Particularly, statins and antiplatelet agents have been shown to effectively reduce the rate of adverse cardiac and limb events. 

In the RECCORD registry, 75% and >80% of patients were on statins and antiplatelet agents, respectively. Though still suboptimal and still need for improvement, the current data of the RECCORD registry are encouraging and a step forward in the right direction when compared to previous observations [14,15,16] with only 30–40% of patients being on statins and 25–30% on antiplatelet agents [14]. The apparent high rate of adherence to guideline-recommended pharmacotherapy in the RECCORD registry might be due to the fact that RECCORD patients are treated at specialized vascular centers. Patients treated at dedicated vascular centers have been shown to receive secondary preventive measures at a higher rate than LEAD patients managed by non-vascular specialists. Therefore, the RECCORD population may not be representative of the wide, general daily practice and hence biased in a favorable manner. It has also previously been reported that secondary preventive pharmacotherapy is inversely related to the severity of LEAD, i.e., CLTI patients had lower prescription rate of statin and antiplatelet agents compared to claudicants [17,18]. In our cohort, we did not find any relation, neither to the severity of diseases, nor to any anatomical segments 

A worrisome finding is the persistent high rate of active smokers (>60% in the aorto-iliac EVR group) despite the well-known deleterious effect of smoking on the progress of LEAD as well as on the adverse long-term outcome following EVR [19,20,21]. The continuing high rate of smokers is a serious lack of secondary prevention which urgently needs to be addressed by health-care providers and patients’ organizations. We did not assess the quality of cardiovascular risk factor control, e.g., how well diabetes, hypertension and hyperlipidemia were controlled according to guidelines-recommended levels. However, previous publications demonstrated the poor control of cardiovascular risk factors in LEAD patients, which is worse than in patients with coronary and cerebrovascular diseases. 

The higher postprocedural rates of DOAC compared to preprocedural rates might mirror the increasing use of DOACs as part of a combined anticoagulation regime according to the COMPASS and VOYAGER trials [22,23].

### 4.5. Strength and Limitations

This study should be interpreted in the context of its observational, descriptive design. Strength of our analysis is its large and comprehensive database of unselected real-world patients, covering the entire spectrum of LEAD without any specific inclusion and exclusion criteria. One potential value of this registry is the provision of data on patients who are generally under-represented in randomized clinical trials such as women, octo- or nanogenerians and patients with extensive comorbidity score including renal and heart failure. 

Our study is limited by the general constraints in the use of register-related health care data as previously described in detail. In a registry-based study like the present one, the responsible clinician has chosen a particular treatment for the individual patient for reasons not encompassed by the concept of the RECCORD registry. As patients are not randomized into different treatment groups, the registry is not designed to compare results between treatment groups. 

Unfortunately, the degree of calcification, which is an important determinant of the lesion complexity, was not assessed in the RECCORD registry. 

We are aware that grouping the treated vessels into four categories might be an over-simplification and that even with a similar extent and level of disease progression, symptoms and their intensity may vary from one patient to another. However, we do not think that a more differentiated analysis of vascular territories would have changed our basic message.

## 5. Conclusions

RECCORD pictures real-world setting of EVR in specialized vascular centers and provides information on several aspects of the daily practice regarding concomitant diseases, endovascular revascularization pattern in relation to anatomic segments, EVR-related complications and use of evidence-based and guidelines-recommended pharmacotherapy in patients with PAD in Germany.

## Figures and Tables

**Table 1:** Baseline demographic and clinical characteristics.

	Aorto-Iliac	Femoropopliteal	Infrapopliteal	Only Infrapopliteal	*p*-Value
Patients ^§^	499 (100.0)	1168 (100.0)	231 (100.0)	95 (100.0)	
Female, *n* (%)	179 (35.9)	407 (34.8)	73 (31.6)	25 (26.3)	
Male, *n* (%)	320 (64.1)	761 (65.2)	158 (68.4)	70 (73.7)	0.247
Age in years, mean ± SD ^§^	66.9 ± 9.9	71.2 ± 9.6	75.9 ± 9.84	76.0 ± 10.9	<0.001
<60 years	120 (24.0)	130 (11.1)	16 (6.9)	9 (9.5)	
60–80 years	338 (67.7)	834 (71.4)	126 (54.5)	45 (47.4)	
>80 years	41 (8.2)	204 (17.5)	89 (38.5)	41 (43.2)	
Body mass index, mean ± SD ^§^	26.1 ± 4.8 (*n* = 465)	26.6 ± 4.9 (*n* = 1080)	26.7 ± 4.7 (*n* = 218)	27.3 ± 5.0 (*n* = 88)	0.115
Hospital length of stay, mean ± SD ^§^	2.9 ± 4.7 (*n* = 465)	3.2 ± 4.2 (*n* = 1073)	6.6 ± 8.3 (*n* = 223)	8.3 ± 8.6 (*n* = 90)	<0.001
Rutherford stages, *n* (%) ^#^	616 (100.0)	1346 (100.0)	248 (100.0)	104 (100.0)	<0.001
Rutherford 0, *n* (%)	2 (0.3)	16 (1.2)	1 (0.4)	0 (0.0)	
Rutherford 1, *n* (%)	42 (6.8)	79 (5.9)	9 (3.6)	4 (3.8)	
Rutherford 2 and 3, *n* (%)	519 (84.3)	952 (70.7)	94 (37.9)	23 (22.1)	
Rutherford 4, *n* (%)	28 (4.5)	113 (8.4)	27 (10.9)	7 (6.7)	
Rutherford 5, *n* (%)	21 (3.4)	149 (11.1)	87 (35.1)	46 (44.2)	
Rutherford 6, *n* (%)	4 (0.6)	37 (2.7)	30 (12.1)	24 (23.1)	
Risk profile and comorbidities ^§^					
Hypertension, *n* (%)	386 (77.4)	991 (84.8)	194 (84.0)	77 (81.1)	0.003
Dyslipidemia, *n* (%)	347 (69.5)	836 (71.6)	145 (62.8)	62 (65.3)	0.044
Diabetes mellitus, *n* (%)	141 (28.3)	418 (35.8)	123 (53.2)	57 (60.0)	<0.001
Active smoking, *n* (%)	321 (64.3)	579 (49.6)	57 (24.7)	16 (16.8)	<0.001
Renal failure, *n* (%)	83 (16.6)	301 (25.8)	89 (38.5)	47 (49.5)	<0.001
Chronic heart failure, *n* (%)	27 (5.4)	97 (8.3)	29 (12.6)	15 (15.8)	0.001
Coronary heart disease, *n* (%)	149 (29.9)	413 (35.4)	97 (42.0)	41 (43.2)	0.004
Cerebrovascular disease, *n* (%)	74 (14.8)	192 (16.4)	43 (18.6)	13 (13.7)	0.537
CAD + Cerebrovascular disease, *n* (%)	38 (7.6)	102 (8.7)	24 (10.4)	6 (6.3)	0.003
Polyneuropathy, *n* (%)	23 (4.6)	72 (6.2)	28 (12.1)	17 (17.9)	<0.001
Previous EVR, *n* (%) ^#^	221 (35.9)	549 (40.8)	102 (41.1)	32 (30.8)	0.051
Previous surgical revascularization, *n* (%) ^#^	51 (8.3)	117 (8.7)	22 (8.9)	8 (7.7)	0.974
Previous amputation, *n* (%) ^§^	10 (2.0)	43 (3.7)	33 (14.3)	17 (17.9)	<0.001

Data are presented as absolute numbers (percentage %) or as mean ± standard deviation. CAD: coronary artery disease; EVR: endovascular revascularization; SD: standard deviation. ^§^ An anatomical vascular segment was counted one-time per patient (*n* = 1898). ^#^ Anatomical vascular segments were counted as many times per patient as recorded in the registry (*n* = 2210).

**Table 2:** Procedural data.

	Aorto-Iliac	Femoropopliteal	Infrapopliteal	Only Infrapopliteal	*p*-Value
Total EVR, *n* (%) ^§^	499 (100.0)	1168 (100.0)	231 (100.0)	95 (100.0)	
EVR segments, *n* (%) ^§^					<0.001
1 segment per EVR	275 (55.1)	917 (78.5)	94 (40.7)	94 (98.9)	
2 or more segments per EVR	224 (44.9)	251 (21.5)	137 (59.3)	1 (1.1)	
EVR site ^§^					<0.001
Left, *n* (%)	197 (39.5)	549 (47.0)	114 (49.4)	52 (54.7)	
Right, *n* (%)	189 (37.9)	612 (52.4)	116 (50.2)	42 (44.2)	
Both, *n* (%)	106 (21.2)	7 (0.6)	1 (0.4)	1 (1.1)	
Other, *n* (%)	7 (1.4)	0 (0.0)	0 (0.0)	0 (0.0)	
Symptoms ^#^					<0.001
Acute, *n* (%)	47 (7.6)	139 (10.3)	33 (13.3)	9 (8.7)	
Chronic, *n* (%)	440 (71.4)	873 (64.9)	93 (37.5)	23 (22.1)	
Acute on chronic, *n* (%)	57 (9.3)	138 (10.3)	12 (4.8)	7 (6.7)	
No specification, *n* (%)	72 (11.7)	196 (14.6)	110 (44.4)	65 (62.5)	
Type of target lesion ^#^					<0.001
Stenosis, *n* (%)	515 (83.6)	732 (54.4)	110 (44.4)	44 (42.3)	
Occlusion, *n* (%)	101 (16.4)	614 (45.6)	138 (55.6)	60 (57.7)	
Target lesion length ^#^					<0.001
<10 cm, *n* (%)	536 (87.0)	573 (42.6)	124 (50.0)	42 (40.4)	
10 cm–20 cm, *n* (%)	67 (10.9)	463 (34.4)	77 (31.0)	42 (40.4)	
>20 cm, *n* (%)	13 (2.1)	310 (23.0)	47 (19.0)	20 (19.2)	
EVR with POBA only, *n* (%) ^#^	51 (8.3)	174 (12.9)	144 (58.1)	66 (63.5)	<0.001
EVR with DCB, *n* (%) ^#^	69 (11.2)	740 (55.0)	48 (19.4)	20 (19.2)	<0.001
1 DCB, *n* (%)	55 (8.9)	466 (34.6)	31 (12.5)	12 (11.5)	
2 DCBs, *n* (%)	10 (1.6)	200 (14.9)	15 (6.1)	7 (6.7)	
3 DCBs, *n* (%)	2 (0.3)	61 (4.5)	2 (0.8)	1 (1.0)	
≥4 DCBs, *n* (%)	2 (0.3)	13 (1.0)	0 (0.0)	0 (0.0)	
EVR with stenting, *n* (%) ^#^	507 (82.3)	610 (45.3)	42 (16.9)	13 (12.5)	<0.001
1 stent, *n* (%)	392 (63.6)	409 (30.4)	31 (12.5)	9 (8.7)	
2 stents, *n* (%)	94 (15.3)	124 (9.2)	5 (2.0)	3 (2.9)	
3 stents, *n* (%)	13 (2.1)	42 (3.1)	2 (0.8)	0 (0.0)	
≥4 stents, *n* (%)	8 (1.3	35 (2.6)	4 (1.6)	1 (1.0)	
EVR with debulking (Atherectomy, rotational thrombectomy), *n* (%) ^#^	12 (1.9)	167 (12.4)	24 (9.7)	10 (9.6)	<0.001

Data are presented as absolute numbers (percentage %). DCB: drug coated balloon; EVR: endovascular revascularization; POBA: plain old balloon angioplasty; SD: standard deviation. ^§^ An anatomical vascular segment was counted one-time per patient (*n* = 1898). ^#^ Anatomical vascular segments were counted as many times per patient as recorded in the registry (*n* = 2210).

**Table 3:** Access site- and procedure-related complications.

	Aorto-Iliac	Femoro-Popliteal	Infra-Popliteal	Only Infrapopliteal	*p*-Value
Total EVR, *n* (%) ^§^	499 (100.0)	1168 (100.0)	231 (100.0)	95 (100.0)	
Total complications, *n* (%) ^§^	16 (3.2)	57 (4.9)	21 (9.1)	8 (8.4)	0.004
Access site related complications, *n* (%) ^§^	9 (1.8)	32 (2.7)	7 (3.0)	4 (4.2)	0.480
Pseudoaneurysm, *n* (%) ^§^	5 (1.0)	26 (2.2)	5 (2.2)	3 (3.2)	
Access site complications requiring surgical/endovascular intervention, *n* (%) ^§^	4 (0.8)	5 (0.4)	2 (0.9)	1 (1.1)	
Bleeding requiring transfusion, *n* (%) ^§^	0 (0.0)	1 (0.1)	0 (0.0)	0 (0.0)	
Procedure-related complications, *n* (%) ^§^	7 (1.4)	29 (2.5)	15 (6.5)	4 (4.2)	<0.001
Distal embolization, *n* (%) ^§^	6 (1.2)	18 (1.5)	12 (5.2)	2 (2.1)	
Device-related complication (dysfunction, displacement), *n* (%) ^§^	0 (0.0)	4 (0.3)	1 (0.4)	0 (0.0)	
Vessel perforation/rupture, *n* (%) ^§^	1 (0.2)	8 (0.7)	3 (1.2)	3 (3.1)	

Data are presented as absolute numbers (percentage %). ^§^ An anatomical vascular segment was counted one-time per patient (*n* = 1898).

**Table 4:** Medication at baseline at the time point of inclusion in the RECCORD registry (pre-EVR) and after the index endovascular revascularization (EVR).

	Aorto-Iliac (*n* = 488)		Femoropopliteal (*n* = 1128)		Infrapopliteal (*n* = 222)		Only Infrapopliteal (*n* = 90)		*p*-Value ^#^
	Pre-EVR	Post-Index EVR	Pre-EVR	Post-Index EVR	Pre-EVR	Post-Index EVR	Pre-EVR	Post-Index EVR	
Lipid lowering agents, *n* (%)	369 (75.6)	438 (89.8)	848 (75.2)	992 (87.9)	170 (76.6)	190 (85.6)	70 (77.8)	76 (84.4)	0.617
ACE inhibitor, *n* (%)	298 (61.1)	302 (61.9)	724 (64.2)	744 (66.0)	145 (65.3)	147 (66.2)	54 (60.0)	55 (61.1)	0.095
Beta-blocker, *n* (%)	229 (46.9)	232 (47.5)	589 (52.2)	598 (53.0)	123 (55.4)	126 (56.8)	55 (61.1)	57 (63.3)	0.010
Antiplatelet therapy, *n* (%)	408 (83.6)	480 (98.4)	940 (83.3)	1104 (97.9)	182 (82.0)	218 (98.2)	73 (81.1)	87 (96.7)	0.719
DOAC, *n* (%)	41 (8.4)	45 (9.2)	159 (14.1)	202 (17.9)	47 (21.2)	60 (27.0)	18 (20.0)	24 (26.7)	<0.001
Vitamin K antagonists, *n* (%)	9 (1.8)	11 (2.3)	39 (3.5)	37 (3.3)	20 (9.0)	20 (9.0)	15 (16.7)	13 (14.4)	<0.001
LMWH, *n* (%)	6 (1.2)	15 (3.1)	14 (1.2)	50 (4.4)	6 (2.7)	21 (9.5)	2 (2.2)	12 13.3)	<0.001
UFH, *n* (%)	0 (0.0)	8 (1.6)	1 (0.1)	23 (2.0)	1 (0.5)	10 (4.5)	1 (1.1)	4 (4.4)	0.017
Oral antidiabetics, *n* (%)	108 (22.1)	102 (20.9)	304 (27.0)	288 (25.5)	93 (41.9)	84 (37.8)	45 (50.0)	43 (47.8)	<0.001

ACE inhibitor: angiotensin-converting-enzyme inhibitor; DOAC: direct oral anticoagulant; LMWH: low molecular weight heparin; pre-EVR: medication before endovascular revascularization; post-index-EVR: medication after the index endovascular revascularization; UFH: unfractionated heparin. ^#^ chi-squared test.

## Data Availability

The data are available from the authors upon reasonable request.
